# Early improvements of individual symptoms as a predictor of treatment response to asenapine in patients with schizophrenia

**DOI:** 10.1002/npr2.12103

**Published:** 2020-03-16

**Authors:** Kamiyu Ogyu, Yoshihiro Noda, Kazunari Yoshida, Shin Kurose, Fumi Masuda, Yu Mimura, Hana Nishida, Eric Plitman, Ryosuke Tarumi, Sakiko Tsugawa, Masataka Wada, Takahiro Miyazaki, Hiroyuki Uchida, Ariel Graff‐Guerrero, Masaru Mimura, Shinichiro Nakajima

**Affiliations:** ^1^ Department of Neuropsychiatry Keio University School of Medicine Tokyo Japan; ^2^ Pharmacogenetic Research Clinic Centre for Addiction and Mental Health Toronto ON Canada; ^3^ Cerebral Imaging Centre Douglas Mental Health University Institute McGill University Montreal QC Canada; ^4^ Multimodal Imaging Group Research Imaging Centre Centre for Addiction and Mental Health University of Toronto Toronto ON Canada

**Keywords:** antipsychotic, asenapine, early improvement, prediction, response, schizophrenia

## Abstract

**Aim:**

It is well accepted that early improvement with antipsychotics predicts subsequent response in patients with schizophrenia. However, no study has examined the contribution of individual symptoms rather than overall symptom severity as the predictors. Thus, we aimed to detect individual symptoms whose improvements could predict subsequent response in patients with schizophrenia during treatment with asenapine and examine whether a prediction model with individual symptoms would be superior to a model using overall symptom severity.

**Methods:**

This study analyzed a dataset including 532 patients with schizophrenia enrolled in a 6‐week double‐blind, placebo‐controlled, randomized trial of asenapine. Response to asenapine was defined as a ≥30% decrease in Positive and Negative Syndrome Scale (PANSS) total score from baseline to week 6. Stepwise logistic regression analyses were performed to investigate the associations among response and PANSS total/individual item score improvements at week 1 or week 2.

**Results:**

Response was associated with early improvement in the following PANSS items: disturbance of volition, active social avoidance, poor impulse control at week 1; and active social avoidance, poor attention, lack of judgment and insight at week 2. Prediction accuracy was almost compatible between the model with individual symptoms and the model with PANSS total score both at weeks 1 and 2 (Nagelkerke *R* ^2^: .51, .42 and .55, .54, respectively).

**Conclusion:**

Early improvement in negative symptoms, poor attention and impulse control, and lack of insight, in particular predicted subsequent treatment response in patients with schizophrenia during treatment with asenapine as accurately as prediction based on overall symptom severity.

## Abstract 2

This study found that treatment response to asenapine was predicted by early improvements of individual symptoms such as negative symptoms, poor attention and impulse control, and lack of insight in patients with schizophrenia. In addition, prediction accuracy was almost comparable between the model with individual symptoms and the model with the PANSS total score, supporting the importance to assess both individual symptoms and the whole severity in the clinical settings.

## Graphical abstract

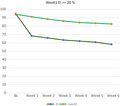

**Abbreviations**
LOCF: last observation carried forward
PANSS: Positive and Negative Syndrome Scale

## 1 INTRODUCTION

Antipsychotics are the mainstay for treatment of schizophrenia.^1^ In particular, second‐generation antipsychotics are commonly used, given that they have favorable profiles, including a decreased risk of neurological side effects compared to first‐generation antipsychotics.^2^ However, second‐generation antipsychotics also have undesirable side effects such as glycolipid metabolism disorders. Therefore, an ineffective course of treatment that carries risks of certain adverse effects should be avoided by predicting antipsychotic treatment response trajectories as early as possible.

Accumulating evidence suggests that an early improvement with antipsychotics serves as a predictor of subsequent response to antipsychotics in patients with schizophrenia,^3^, ^4^, ^5^ with the greatest symptom reduction occurring within the first week of the antipsychotic initiation.^6^ However, previous studies targeting schizophrenia have investigated the relationship between overall severity of symptoms and subsequent treatment response,^3^, ^4^, ^5^ focusing on the sum of the scores in the representative rating scales (eg, the Positive and Negative Syndrome Scale (PANSS)). To the best of our knowledge, no study to date has investigated whether early improvements in individual symptoms could predict subsequent response to antipsychotics in this population except for one study. More specifically, Ruberg et al used classification and regression tree (CART) analysis to identify individual positive symptoms whose early changes can predict long‐term response to atypical antipsychotics in the treatment of schizophrenia.^7^ In fact, modern psychiatry is limited in its evaluation of clinical severity by a total score rather than measurable individual scores. Thus, it is crucial to detect individual symptoms whose improvements can predict subsequent response for each patient with schizophrenia in clinical practice.

To fill in the gap in the literature, we utilized clinical data (P06124 trial: a 6‐week randomized, double‐blind, placebo‐controlled trial of the effectiveness of asenapine in participants with an acute exacerbation of schizophrenia) in order to test the following hypotheses: (1) Early improvement in individual symptoms will predict subsequent response to asenapine in patients with schizophrenia and (2) the prediction model with individual symptoms will be more accurate than the model with overall symptom severity.

## 2 MATERIALS AND METHODS

### 2.1 Study design

Following a complete description of the study, subjects provided written informed consent at study enrollment in the original study, which was approved by the institutional review board of all participating sites. This post hoc analysis was made completely anonymous; thus, ethical approval was not sought out for this study. This study analyzed data from a 6‐week multicenter, randomized, double‐blind, placebo‐controlled phase III trial of asenapine conducted from May 2010 to April 2014 in Japan, Republic of Korea, and Taiwan. The original study consisted of a screening phase (ie, 3‐7 days from the screening test to the baseline), 6‐week treatment period, and follow‐up phase. During the screening period, patients were administered placebo tablets twice a day in a single‐blind manner to exclude patients with high placebo responsiveness, as described below. This study enrolled males and females, aged 20‐65 years, having a diagnosis of schizophrenia with an acute exacerbation based on the DSM‐IV‐TR criteria. Patients were selected based on the following criteria at baseline of the treatment phase: (a) assessed at the screening phase, (b) a score ≥60 on PANSS total score at baseline, (c) scores of ≥4 on two or more items on the PANSS positive symptom subscale (delusion, conceptual disorganization, hallucinatory behavior, excitement, grandiosity, suspiciousness, or hostility), and (d) a score ≥4 on the Clinical Global Impressions—severity Illness scale at baseline. Based on the results obtained from the short‐term administrations of phase II and phase III in the original study,^8^ the current episode period was set to be within 2 months. To exclude patients with high placebo response, those who had a score reduction of ≥20% on the PANSS total score during the screening phase were excluded.^8^

After baseline assessment was completed, patients considered to be eligible by the investigator were randomized (1:1: 1) to receive sublingual asenapine 5 mg bid, 10 mg bid, or placebo. During the study period, patients received asenapine or placebo doses twice daily for 6 weeks. The tablets were administered sublingually without water. The PANSS assessment was completed at baseline and weekly through week 6. Study physicians, patients, and raters were all blinded throughout the study.

### 2.2 Statistical analysis

We used the dataset for the last observation carried forward (LOCF) analysis, in which the score at the previous visit was adopted in the case of premature attrition. The Shapiro‐Wilk test was performed to test the normality of the data. As a result, we found that the data did not follow the normal distribution. Baseline socio‐clinico‐demographic characteristics were compared between responders and non‐responders by Mann‐Whitney *U* test and chi‐square or Fisher's test for continuous variables and categorical variables, respectively. We employed baseline sociodemographic and clinical characteristics that were statistically different between the 2 groups and the changes of each individual symptom from week 0 to week 1 or week 2 as the independent variables. Percent decrease in PANSS total scores was calculated as (PANSS total scores at baseline − PANSS total scores at week 6) × 100/(PANSS total scores at baseline − 30). Here, based on previously published studies,^8^ response to antipsychotics was defined as a ≥30% decrease in PANSS total scores. Based on the previous studies,^5^ we defined early improvement as 20% decreases in PANSS total scores at week 2. Stepwise logistic regression analyses were performed to evaluate the relationships among early improvements of PANSS total scores at week 1 or week 2 and treatment response at week 6. We explored sequentially defining early improvement as 5%, 10%, 15%, and 20% decreases in PANSS total scores at week 1 or week 2. Stepwise logistic regression analyses were also performed to evaluate the relationships among changes of PANSS total scores at week 1 or week 2 and treatment response at week 6. We employed two definitions of early improvement with categorical and continuous data of early improvement for these analyses as independent variables. Although these two approaches are complementary to each other, the latter method was applied to confirm more detailed changes in the early response. Finally, as a primary analysis, stepwise logistic regression analyses were performed to evaluate the relationships among early improvements of PANSS individual item scores at week 1 or week 2 and treatment response at week 6. Sensitivity, specificity, and accuracy for the prediction models were calculated for the analyses on overall symptoms and individual symptoms, respectively. A *P*‐value of <.05 was considered statistically significant (2‐sided). Statistical analyses were performed with IBM SPSS Statistics version 24.0 (IBM Japan).

## 3 RESULTS

### 3.1 Clinico‐demographic characteristics of the patients

Clinico‐demographic characteristics of 315 patients are summarized in Table 1. Almost half of the patients were Japanese and one third were Taiwanese, aged 41.5 ± 11.0 in the responder group, and 41.6 ± 11.1 in the non‐responder group, with a nearly equal male‐female ratio. Responders were associated with higher rates of comorbid psychiatric diseases and lower rates of Korean patients, dropouts, and intake of anticholinergics in comparison with non‐responders (Table 1).

**TABLE 1:** Clinico‐demographic characteristics of the subjects

Characteristics	Responder (n = 78）	Non‐responder (n = 239)	Statistics
	Mean ± SD or n (%)	Mean ± SD or n (%)	
PANSS total score at baseline	94.1 (18.3)	94.3 (17.9)	*U* = 9192, *Z* = −0.18, *P* = .86
**PANSS total score at week 1**	**77.5 (17.2)**	**91.9 (21.7)**	***U* = 5440, *Z* = −5.27, *P* < .001**
**PANSS total score at week 2**	**70.9 (16.4)**	**90.4 (22.6)**	***U* = 4346, *Z* = −6.85, *P* < .001**
PANSS positive scale	23.6 (4.6)	24.3 (5.2)	*U* = 8675, Z =−0.92, *P* = .36
PANSS negative scale	23.1 (5.7)	23.9 (6.3)	*U* = 8707, *Z* = −0.88, *P* = .38
PANSS general psychopathology scale	47.2 (10.8)	46.2 (10.0)	*U* = 8839, *Z* = −0.68, *P* = .49
Age	41.5 (11.0)	41.6 (11.1）	*U* = 9268, *Z* = −0.07, *P* = .94
Weight	65.0 (13.9)	62.9 (13.9)	*U* = 8577, *Z* = −1.06, *P* = .29
BMI	24.2 (4.3)	23.8 (4.3)	*U* = 8908, *Z* = −0.59, *P* = .56
Age of onset	28.9 (9.2)	27.6 (9.4)	*U* = 8492, *Z* = −1.18, *P* = .24
Duration period	13.0 (10.0)	14.5 (11.0)	*U* = 8621, *Z* = −0.10, *P* = .32
Dose of asenapine			*χ* ^2^ = 1.45, *df* = 1, *P* = .23
10mg	43 (27.6)	113 (72.4)	
5mg	35 (21.7)	126 (78.3)	
**Dropout rate**	**7 (9.0)**	**87 (36.4)**	***χ*^2^ = 21.21, *df* = 1, *P* < .001**
**Country**	***χ*^2^ = 9.07, df = 2, J vs T: *P* = .383, J vs K: *P* = .011, K vs T: *P* = .003**		
Japan (J)	44 (56.4)	125 (52.3)	
Taiwan (T)	28 (35.9)	61 (25.5)	
Korea (K)	6 (7.7)	53 (22.2)	
Male	35 (44.8)	121 (51.3)	*χ* ^2^ = 0.78, *df* = 1, *P* = .43
Family history of diabetes mellitus	16 (20.5)	29 (12.1)	*χ* ^2^ = 4.03, *df* = 2, *P* = .13
Family history of coronary diseases	11 (14.1)	15 (6.3)	*χ* ^2^ = 5.54, *df* = 2, *P* = .06
Complication	6 (7.7)	30 (12.6)	*χ* ^2^ = 1.38, *df* = 1, *P* = .31
Duration of this episode			*χ* ^2^ = 3.48, *df* = 3, *P* = .54
≤8 y	0 (0)	1 (0.4)	
4‐8 y	44 (56.4)	114 (47.7)	
2‐4 y	23 (29.5)	80 (33.5)	
>2 y	11 (14.1)	44 (18.4)	
Number of episodes needed hospitalization			*χ* ^2^ = 3.97, *df* = 4, *P* = .41
0	16 (20.5)	37 (15.5)	
1	16 (20.5)	44 (18.4)	
2‐3	22 (28.2)	55 (23.0)	
more than 4	21 (26.9)	88 (36.8)	
unknown	3 (3.8)	15 (6.3)	
**Presence of other psychiatric diseases**	**10 (12.8)**	**5 (2.1)**	***χ*^2^ = 15.01, *df* = 1, *P* = .001**
Education year			*χ* ^2^ = 4.62, *df* = 3, *P* = .23
12≤	20 (25.6)	49 (20.5)	
6‐12	50 (64.1)	173 (72.4)	
6>	7 (9.0)	17 (7.1)	
unknown	1 (1.3)	0 (0)	
Presence of pretreatment antipsychotic	74 (94.9)	216 (90.4)	*χ* ^2^ = 1.53, *df* = 1, *P* = .25
**Using anticholinergic**	**17 (21.8)**	**87 (36.4)**	***χ*^2^ = 5.69, *df* = 1, *P* = .02**

Abbreviation: PANSS, Positive and Negative Syndrome Scale.

Bold letters indicate significance (*P* < .05).

### 3.2 Prediction with overall symptom severity

**FIGURE 1:**
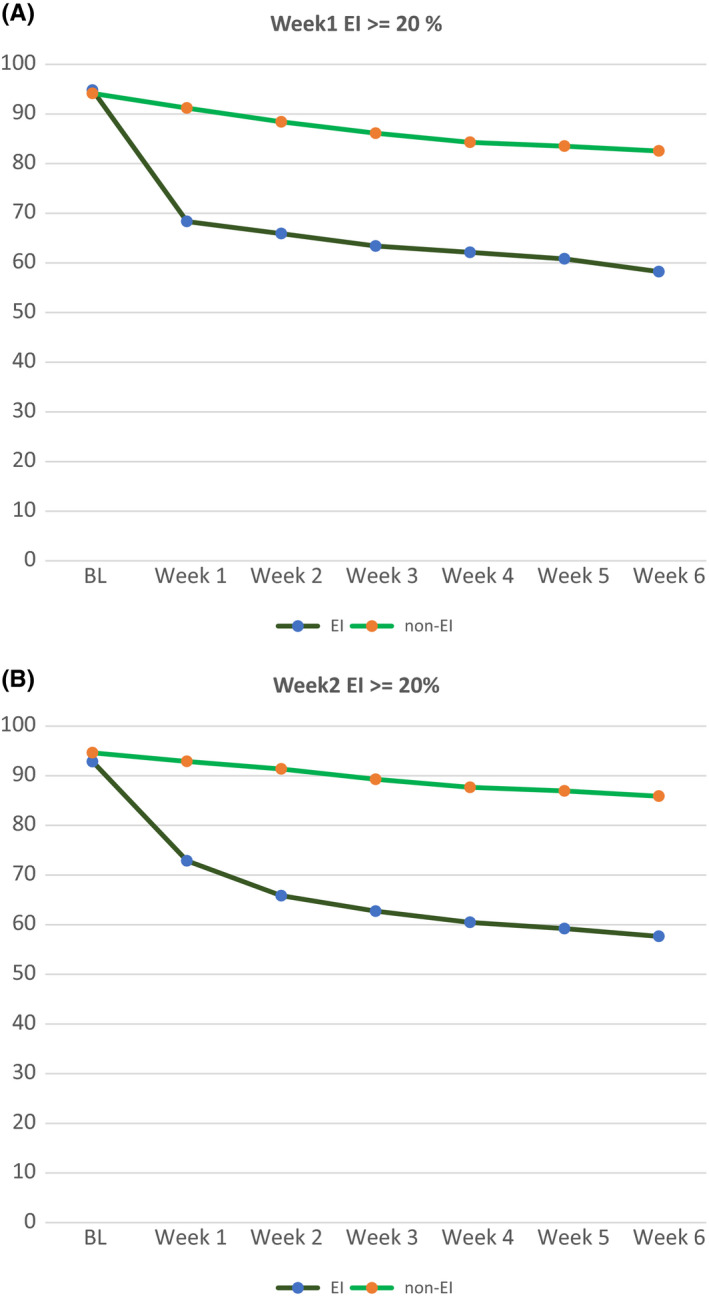
A, Trajectories of PANSS scores for 20% improvers and non‐improvers in week 1. B, Trajectories of PANSS scores for 20% improvers and non‐improvers in week 2. EI; early improvement, non‐EI; non‐early improvement, PANSS; Positive and Negative Syndrome Scale

Tables 2 and 3 summarizes the number of patients who showed early improvement at week 1 and week 2, and the number of those who responded at week 6. Figure 1 shows the PANSS total scores for responders and non‐responders, at week 1 and week 2 in the early improvement group and the early non‐improvement group, respectively. The logistic regression analysis with early improvement of 20% as a categorical variable showed significant results (*χ* ^2^ = 106.7, *df* = 5, *P* < .0001 at week 1, and *χ* ^2^ = 142.7, *df* = 7, *P* < .0001 at week 2) which explained 43.0% and 54.5% (Nagelkerke *R* ^2^) of the variance in responders with improvements at weeks 1 and 2, respectively. The prediction performance of binary classification of early improvement at weeks 1 and 2 for response at week 6 is shown in Tables 4 and 5. The 20% cutoff in the PANSS at week 2 showed the highest degree of accuracy for predicting response at week 6 (Tables 4 and 5). Also, the logistic regression analysis with score reductions in the PANSS total score as a continuous variable showed significant results (*χ* ^2^ = 111.6, *df* = 3, *P* < .0001 at week 1, and *χ* ^2^ = 149.6, *df* = 5, *P* < .0001 at week 2) which explained 44.6% and 56.5% (Nagelkerke *R* ^2^) of the variance in responders with improvements at weeks 1 and 2, respectively.

**TABLE 2:** Numbers of those with 20% improvement in week 1 and those with responder in week 6

	Non‐responder	Responder
Non‐early improvement	216	23
Early improvement	28	48

**TABLE 3:** Numbers of those with 20% improvement in week 2 and those with responder in week 6

	Non‐responder	Responder
Non‐early improvement	214	25
Early improvement	23	53

**TABLE 4:** Prediction performance of score reduction at week 1 for response at week 6

	Early improvement in week 1 (%）			
	5	10	15	20
Sensitivity	0.43	0.42	0.66	0.63
Specificity	0.89	0.92	0.87	0.90
Accuracy	0.78	0.80	0.82	0.84

**TABLE 5:** Prediction performance of score reduction at week 2 for response at week 6

	Early improvement in week 2 (%）			
	5	10	15	20
Sensitivity	0.12	0.49	0.55	0.70
Specificity	0.98	0.89	0.90	0.90
Accuracy	0.78	0.79	0.82	0.85

### 3.3 Prediction with individual symptom severity

**FIGURE 2:**
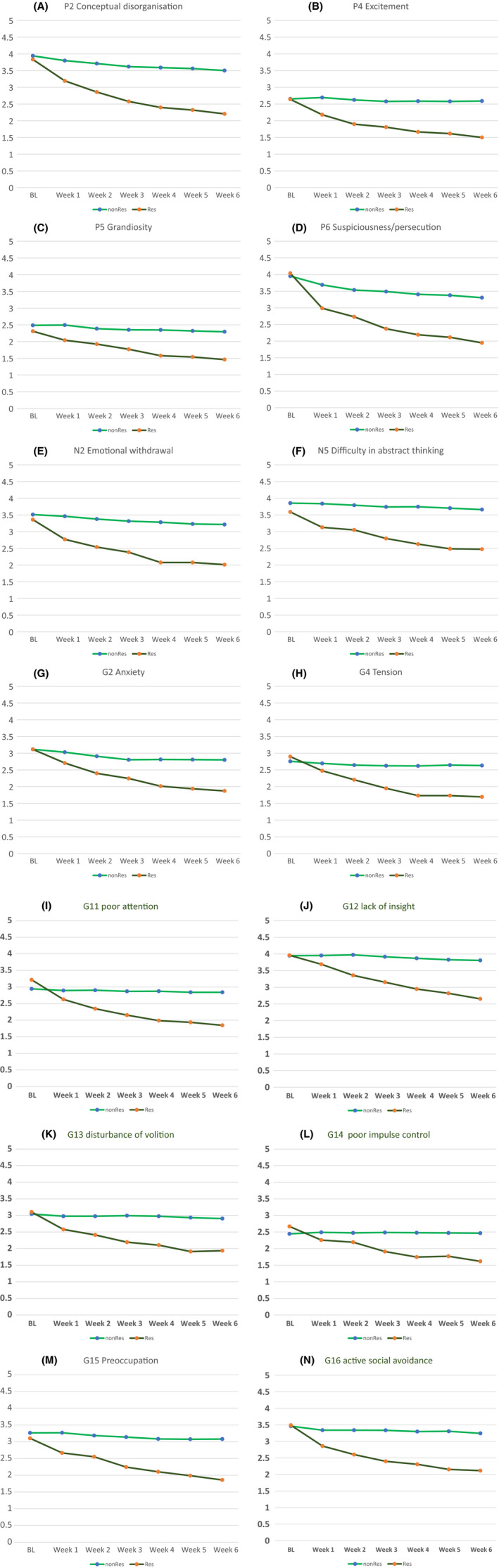
A, Trajectories of individual symptoms in the PANSS score (P2 conceptual disorganisation). B, Trajectories of individual symptoms in the PANSS score (P4 excitement). C, Trajectories of individual symptoms in the PANSS score (P5 grandiosity). D, Trajectories of individual symptoms in the PANSS score (P6 suspiciousness/persecution). E, Trajectories of individual symptoms in the PANSS score (N2 emotional withdrawal). F, Trajectories of individual symptoms in the PANSS score (N5 difficulty in abstract thinking). G, Trajectories of individual symptoms in the PANSS score (G5 anxiety). H, Trajectories of individual symptoms in the PANSS score (G4 tension). I, Trajectories of individual symptoms in the PANSS score (G11 poor attention). J, Trajectories of individual symptoms in the PANSS score (G12 lack of insight). K, Trajectories of individual symptoms in the PANSS score (G13 disturbance of volition). L, Trajectories of individual symptoms in the PANSS score (G14 poor impulse control). M, Trajectories of individual symptoms in the PANSS score (G15 preoccupation). N, Trajectories of individual symptoms in the PANSS score (G16 active social avoidance). PANSS; Positive and Negative Syndrome Scale

Response at week 6 was significantly associated with improvements at weeks 1 and 2 in the PANSS items: disturbance of volition, active social avoidance, poor impulse control, emotional withdrawal, suspiciousness, tension, and difficulty in abstract thinking at week 1, and active social avoidance, poor attention, lack of judgment and insight, grandiosity, preoccupation, anxiety, excitement, emotional withdrawal, and conceptual disorganization at week 2, in this order (Table 6; Figure 2). The logistic regression analysis showed significant results (*χ* ^2^ = 126.7, *df* = 12, *P* < .0001 with improvements at week 1 and *χ* ^2^ = 140.7, *df* = 10, *P* < .0001 with improvements at week 2). The model explained 49.7% and 54.0% (Nagelkerke *R* ^2^) of the variance and correctly classified 83.1% and 85.7% of responders with improvements at weeks 1 and 2, respectively. Sensitivity, specificity, PPV, and NPV were 70.9%, 85.7%, 51.3%, and 93.2%, respectively, at week 1 and 75.4%, 88.1%, 60.5%, and 93.7%, respectively, at week 2 (Table 7).

**TABLE 6:** Association between early improvements at week 1 in individual symptoms in the PANSS and subsequent response in patients with schizophrenia

	*χ* ^2^ = 126.7, *df* = 12, *P* < .0001, Nagelkerke *R* ^2^ = .50, sensitivity = 0.51, specificity = 0.93, accuracy = 0.83					
	Individual symptom	SE	*P* value	Odds ratio	95%CI	
P1	Delusions					
P2	Conceptual disorganization					
P3	Hallucinatory behavior					
P4	Excitement					
P5	Grandiosity					
P6	Suspiciousness/persecution	−0.55	.01	0.58	0.39	0.86
P7	Hostility					
N1	Blunted affect					
N2	Emotional withdrawal	−0.54	.02	0.58	0.38	0.90
N3	Poor rapport					
N4	Passive/apathetic social withdrawal					
N5	Difficulty in abstract thinking	−0.73	.003	0.48	0.29	0.78
N6	Lack of spontaneity and flow of conversation					
N7	Stereotyped thinking					
G1	Somatic concern					
G2	Anxiety					
G3	Guilt feelings					
G4	Tension	−0.63	.004	0.53	0.35	0.82
G5	Mannerisms and posturing					
G6	Depression					
G7	Motor retardation					
G8	Uncooperativeness					
G9	Unusual thought content					
G10	Disorientation					
G11	Poor attention					
G12	Lack of judgment and insight					
G13	Disturbance of volition	0.74	.004	2.10	1.27	3.47
G14	Poor impulse control	0.62	.02	1.86	1.10	3.14
G15	Preoccupation					
G16	Active social avoidance	0.68	.01	1.98	1.21	3.23

Abbreviations: PANSS, Positive and Negative Syndrome Scale; SE, standard error.

**TABLE 7:** Association between early improvements at week 2 in individual symptoms in the PANSS and subsequent response in patients with schizophrenia

	*χ* ^2^ = 140.7, *df* = 10, *P* < .0001, Nagelkerke *R* ^2^ = .54, sensitivity = 0.61, specificity = 0.94, accuracy = 0.86					
	Individual symptom	SE	*P* value	Odds ratio	95%CI	
P1	Delusions					
P2	Conceptual disorganization	−0.69	.004	0.50	0.31	0.81
P3	Hallucinatory behavior					
P4	Excitement	−0.55	.01	0.58	0.38	0.87
P5	Grandiosity	−0.48	.04	0.62	0.39	0.97
P6	Suspiciousness/persecution					
P7	Hostility					
N1	Blunted affect					
N2	Emotional withdrawal	−0.63	.01	0.53	0.34	0.84
N3	Poor rapport					
N4	Passive/apathetic social withdrawal					
N5	Difficulty in abstract thinking					
N6	Lack of spontaneity and flow of conversation					
N7	Stereotyped thinking					
G1	Somatic concern					
G2	Anxiety	−0.51	.02	0.60	0.40	0.90
G3	Guilt feelings					
G4	Tension					
G5	Mannerisms and posturing					
G6	Depression					
G7	Motor retardation					
G8	Uncooperativeness					
G9	Unusual thought content					
G10	Disorientation					
G11	Poor attention	0.81	.001	2.24	1.40	3.59
G12	Lack of judgment and insight	0.57	.02	1.76	1.11	2.79
G13	Disturbance of volition					
G14	Poor impulse control					
G15	Preoccupation	−0.50	.05	0.61	0.37	0.10
G16	Active social avoidance	0.95	.001	2.59	1.60	4.19

Abbreviations: PANSS, Positive and Negative Syndrome Scale; SE, standard error.

## 4 DISCUSSION

This is the first study to examine whether early improvement of individual symptoms including negative symptoms and cognitive impairment could serve as clinically useful predictors of response in patients with schizophrenia during an acute course of treatment. We found that improvements of the following symptoms in the PANSS score at weeks 1 and 2 were related to treatment response to asenapine at week 6: disturbance of volition, active social avoidance, and poor impulse control at week 1; active social avoidance, poor attention, and lack of judgment and insight at week 2.

Ruberg et al conducted a similar retrospective study to identify predictors of treatment response that most effectively differentiated responders from non‐responders in chronic patients with schizophrenia by using CART analysis. They found that improvement of six positive symptom items in the PANSS two weeks after antipsychotic administration predicted treatment response after eight weeks. However, there are some differences in the methodology between their study and ours. First, they used mixed datasets from multiple clinical studies while we used data from a single phase III trial of asenapine. Second, Ruberg's study was limited to chronic patients with schizophrenia who had a long duration of illness while this study targeted those in the acute exacerbation phase. Lastly, despite the similar aims between them, Ruberg et al examined the relationship with the CART analysis, focusing only on the PANSS subscale items of positive symptoms. In contrast, we examined the relationships using all of the subscale items of PANSS to identify predictors of early response by asenapine. To the best of our knowledge, no other studies to date have investigated the relationships between early improvement in all individual symptoms and subsequent response in patients with schizophrenia during antipsychotic treatment, while a few studies reported that improvements in several individual symptoms in the early stages of treatment are related to subsequent treatment response in patients with depression.^9^, ^10^ We also noted that early improvements of overall symptom severity by asenapine predicted subsequent treatment response in patients with schizophrenia, which is in line with previous studies that have demonstrated that early improvement in overall severity at week 2 was associated with subsequent response to antipsychotics in patients with schizophrenia.^3^, ^4^, ^5^ Notably, with regard to the Nagelkerke *R* ^2^ power and case classification rates, prediction accuracy was compatible between the model with early improvement of individual symptoms and the model with early improvement of the PANSS total score both at weeks 1 and 2.

### 4.1 Negative symptoms

In this study, early improvement in active social avoidance, a secondary negative symptom, defined as the restriction of social relationships with unfounded fear, hostility, and distrust, was the strongest predictor at week 2 and the second strongest one at week 1 for treatment response to asenapine at week 6. Moreover, the most relevant predictor at week 1 was the disturbance of volition, which is a primary negative symptom defined as an obstacle to voluntary start, duration, and maintenance in thought, movement, behavior, and conversation. A most recent meta‐analysis noted that multiple treatments including second‐generation antipsychotics (ES = 0.58), psychotherapy (ES = 0.40), and antidepressants (ES = 0.35) were found effective for negative symptoms.^11^ Although this meta‐analysis demonstrated that there was no clinical effectiveness of antipsychotics on negative symptoms, we speculate that their therapeutic effect for negative symptoms might be heterogeneous depending on subgroups or individuals within this population. On the other hand, negative symptoms can be divided into primary and secondary negative symptoms, where the latter refers to all symptoms that look like negative symptoms caused by positive symptoms, anxiety, depression, chronic abuse of illicit drugs and alcohol, oversedation by high dosage of antipsychotics, social deprivation, lack of stimulation, and hospitalization.^12^ As such, it is important to distinguish between primary and secondary negative symptoms because the latter might be treatable while the treatment has not yet been fully established for the former, a key part of the symptomatology of schizophrenia. However, it still remains unclear whether antipsychotics are effective for “secondary” negative symptoms exclusively or also “primary” negative symptoms.^13^ To date, very few clinical trials have examined the effectiveness of antipsychotics focusing on schizophrenia patients with only primary negative symptoms, which has resulted in mixed results due to the heterogeneity of this disorder.^12^ Given our results, therefore, it is crucial to identify individuals with schizophrenia who show early improvement in avolition and active social avoidance in an effort to predict their subsequent response to asenapine.

### 4.2 Cognitive dysfunction

In this study, early improvement in poor attention was the second strongest predictor at week 1 for treatment response to asenapine at week 6. Poor attention is a component of cognitive impairment, defined as the difficulty of switching, holding, and setting attention to new stimuli due to internal/external stimuli.^14^ Like negative symptoms, cognitive impairment is among the strongest predictors associated with functional prognosis.^15^ One previous meta‐analysis reported that patients with schizophrenia have poor attention, within an ES = 0.20‐0.40.^16^ Nielsen et al^17^ demonstrated that there is no effect of antipsychotics on attention. Despite the limited effects by asenapine on cognitive impairment including poor attention, our finding suggests that it is necessary to pay careful attention not only to positive symptoms but also to cognitive dysfunction in the acute phase although the causality between early improvement of attention and treatment response remains unknown. Given the lack of procognitive drugs for patients with schizophrenia,^18^, ^19^ further research is clearly warranted to examine the neural basis of cognitive dysfunction and facilitate the development of treatments such as neuromodulation based on the pathophysiology of this disorder.^20^

### 4.3 Poor impulse control

Early improvement in poor impulse control was the third strongest predictor at week 1 for treatment response to asenapine at week 6. Poor impulse control, a factor of excited component of the PANSS,^14^ is a significant clinical and social problem in patients with schizophrenia, leading to increased risk of violence, complications during hospitalization, and prolongation of hospital stay.^21^, ^22^ Therefore, it is important to improve impulsivity in patients with schizophrenia during an earlier treatment phase. Indeed, it is noted that antipsychotics, including asenapine, cause an early improvement in impulsivity in this population. Studies investigating the effects of asenapine on excitement in patients including those with schizophrenia showed a significant decrease in the PANSS excited score 2 hours after asenapine treatment (NNT 3 (95% CI 2‐4)).^23^ In addition, Volavka et al^24^ showed that olanzapine improved hostility, which is another excitatory component, at an early treatment phase. Thus, these findings suggest that optimal antipsychotic treatment improves poor impulse control in patients with schizophrenia, which may in turn lead to subsequent favorable treatment outcomes in this population.

### 4.4 Lack of insight

It is known that more than 50% of patients with schizophrenia present with moderate‐to‐severe lack of insight. Lack of insight in schizophrenia is associated with low medication adherence and poor outcomes leading to relapse and high mortality.^25^, ^26^ We found that early improvement in lack of judgment and insight was the third strongest predictor at week 2 for treatment response to asenapine at week 6. In support, previous studies demonstrated that lack of insight is associated with overall disease severity as well as cognitive dysfunction and depressive symptoms in this population.^26^, ^27^, ^28^, ^29^ Therefore, early improvement in lack of insight by treatment with asenapine may improve medication adherence in patients with schizophrenia, which in turn leads to treatment response in this population. Furthermore, a recent meta‐analysis analyzing 14 RCTs reported that antipsychotics significantly improved lack of insight (ES = 0.23) in the first 6 weeks compared with placebo in patients with schizophrenia and that improvement of lack of insight was also related to improvements of other PANSS sub‐scores.^30^ These findings appear consistent with our results that early improvement in lack of judgment and insight could be a predictor of subsequent treatment response to asenapine. Given that the effect of antipsychotics on lack of insight is limited, non‐invasive neuromodulation may be useful for accelerating early improvement in lack of insight in patients with schizophrenia.^31^ Therefore, further research is warranted to elucidate the biological mechanism of lack of insight and to develop therapeutic methods such as neuromodulation based on the corresponding neural processes.

### 4.5 Limitations

The results of our study must be interpreted in light of some limitations. First, the P06124 trial was not originally designed to test our hypotheses. Second, the generalizability of our findings may be limited to Japanese, Korean, Taiwanese patients with schizophrenia treated with asenapine. Third, the diagnosis of schizophrenia according to DSM‐IV‐TR criteria includes heterogeneous populations in terms of clinical features. Fourth, we found that the dropout rate was significantly higher in non‐responder group. Last observation carried forward analysis assumes that patients maintain clinical symptom scores at the time of dropout up to the end of the study. Thus, the significant difference in the dropout rate between the group may affect the predictive models using LOCF method. Fifth, we could not examine primary and secondary negative symptoms because the original data did not strictly distinguish between them. Sixth, since the predictive value of individual PANSS score has not been verified by another test dataset, it is necessary to use another cohort data to verify the reproducibility of this research result. Seventh, in order to examine the true antipsychotic effect of asenapine by excluding placebo responders, the original study performed the screening period with placebo. Still, we found that early improvement with asenapine predicted subsequent treatment response, suggesting that placebo response may not contribute to the predictor role of early improvement. Eighth, the sample size is large enough to detect minor effect. Most significant individual symptom changes are less than 1 point in the first or second week, which is of limited importance in clinical practice. Finally, two different dose groups (5, 10 mg/day) were all treated as the asenapine group. Although it may have been ideal to include dose information in our analysis in light of the possible dose‐response relationship of asenapine, there was no statistical difference in the asenapine dose between responders and non‐responders.

## 5 CONCLUSIONS

This study found that treatment response to asenapine was predicted by early improvements of individual symptoms such as negative symptoms, poor attention and impulse control, and lack of insight in patients with schizophrenia. Moreover, prediction accuracy was almost comparable between the model with individual symptoms and the model with the PANSS total score, supporting the importance to assess both individual symptoms and the whole severity in the clinical settings. Notably, there is little evidence for switching antipsychotics when patients with schizophrenia show insufficient response to one antipsychotic.^32^ Furthermore, it was noted that there is a great delay for initiating clozapine for patients with treatment‐resistant schizophrenia.^33^ Therefore, further studies are warranted to explore predictors of antipsychotic treatment response, which can be utilized for switching from first‐line antipsychotics to clozapine at the earliest plausible timing in this population.

## CONFLICT OF INTEREST

Dr Ogyu has received grants from Inokashira Hospital Research Fund. Dr Noda has received an investigator‐initiated clinical study grant from TEIJIN PHARMA LIMITED and research grants from Japan Health Foundation, Meiji Yasuda Mental Health Foundation, Mitsui Life Social Welfare Foundation, Takeda Science Foundation, SENSHIN Medical Research Foundation, Health Science Center Foundation, Mochida Memorial Foundation for Medical and Pharmaceutical Research, and Daiichi Sankyo Scholarship Donation Program. He receives equipment‐in‐kind supports for an investigator‐initiated study from Magventure Inc, Inter Reha Co., Ltd, Rogue Resolutions Ltd, and Miyuki Giken Co., Ltd. Dr Yoshida has received manuscript fees from Sumitomo Dainippon Pharma, fellowship grants from the Japan Research Foundation for Clinical Pharmacology, and consultant fees from Bracket and VeraSci within the past 3 years. Dr Graff‐Guerrero has received support from the United States National Institute of Health, CIHR, OMHF, Consejo Nacional de Ciencia y Tecnología, the Instituto de Ciencia y Tecnología del DF, the Brain & Behavior Research Foundation (Formerly NARSAD), the Ontario Ministry of Health and Long‐Term Care, the Ontario Ministry of Research and Innovation Early Research Award, and Janssen. Dr Uchida has received grants from Eisai, Otsuka Pharmaceutical, Dainippon‐Sumitomo Pharma, Mochida Pharmaceutical, Meiji‐Seika Pharmaceutical, and Novartis; speaker's honoraria from Otsuka Pharmaceutical, Eli Lilly, Shionogi, Pfizer, Yoshitomi Yakuhin, Dainippon‐Sumitomo Pharma, Meiji‐Seika Pharma, MSD, and Janssen Pharmaceutical; and advisory panel payments from Dainippon‐Sumitomo Pharma within the past 3 years. Dr Mimura has received grants or speaker's honoraria from Asahi Kasei Pharma, Astellas Pharmaceutical, Daiichi Sankyo, Dainippon‐Sumitomo Pharma, Eisai, Eli Lilly, GlaxoSmithKline, Janssen Pharmaceutical, Meiji‐Seika Pharma, Mochida Pharmaceutical, MSD, Novartis Pharma, Otsuka Pharmaceutical, Pfizer, Shionogi, Takeda, Tanabe Mitsubishi Pharma, and Yoshitomi‐Yakuhin within 3 years. Dr Nakajima has received fellowship grants from CIHR, research support from Japan Society for the Promotion of Science, Japan Research Foundation for Clinical Pharmacology, Naito Foundation, Uehara Memorial Foundation, Takeda Science Foundation, Daiichi Sankyo, and MSD, manuscript fees or speaker's honoraria from Dainippon Sumitomo Pharma and Yoshitomi Yakuhin within the past 3 years. Other authors have no financial or other relationship relevant to the subject of this manuscript.

## AUTHOR CONTRIBUTION

S. Nakajima, Y. Noda, and K. Ogyu were responsible for the study concept and design. S. Nakajima, Y. Noda, M. Wada, and K. Ogyu were responsible for statistical analysis. S. Nakajima, Y. Noda, K. Yoshida, and K. Ogyu were responsible for drafting the manuscript. All other authors were responsible for critical revision of the manuscript and have accepted the final version. All other authors contributed to and have approved the final manuscript.

## Data Availability

We obtained the data that support the findings of this study from Meiji Seika Pharma with a contract between us, which defined the group that can use the data and the duration for which the data is usable. Data are available from Meiji Seika Pharma upon reasonable request to Meiji Seika Pharma.
